# Alterations in executive functions in inmates convicted for violent behavior: a systematic review

**DOI:** 10.3389/fpsyg.2023.1066474

**Published:** 2023-08-16

**Authors:** Maria Antonia Chaguendo-Quintero, Daniela Quintero-Monjes, Maria Teresa Cuervo, Juan P. Sanabria-Mazo

**Affiliations:** ^1^Department of Social Sciences, Pontificia Universidad Javeriana, Cali, Colombia; ^2^Teaching, Research, and Innovation Unit, Parc Sanitari Sant Joan de Dèu, Barcelona, Spain; ^3^Centre for Biomedical Research in Epidemiology and Public Health (CIBERESP), Madrid, Spain; ^4^Department of Basic, Developmental, and Educational Psychology, Universidad Autónoma de Barcelona, Barcelona, Spain

**Keywords:** inmates, violent behavior, executive functions, inhibition, cognitive flexibility, working memory, systematic review

## Abstract

**Background:**

The growth of the prison population and the high recidivism rates of inmates represent a major public safety problem.

**Objective:**

This systematic review explored executive functions in inmates convicted of violent behavior compared with inmates convicted of non-violent behavior and healthy controls (HCs).

**Methods:**

Systematic searches were carried out using five databases (PubMed, Scopus, Web of Science, EBSCO, and Embase) until March 6th, 2023. Following Preferred Reporting Items for Systematic Reviews and Meta-analyses (PRISMA) guidelines, two reviewers independently performed the screening, data extraction, and risk of bias assessment of the 8 studies included. The protocol of this study was registered in Prospective Register of Systematic Reviews (PROSPERO), under registration number CRD42021252043.

**Results:**

Consistently, inmates convicted of violent behavior exhibited a greater alteration in inhibition than inmates convicted of non-violent behavior (four out of four studies) and HCs (two out of two studies). In addition, inmates convicted of violent behavior showed greater impairments in cognitive flexibility (two out of three studies) and working memory (two out of three studies) than HCs. Although with limited evidence (only one study), they also showed worse performance in set shifting and planning than HCs.

**Conclusion:**

This study provides evidence of alterations in inhibition in inmates convicted of violent behavior compared to inmates convicted of non-violent behavior and HCs. Even though inmates convicted of violent behavior showed greater impairments in planning and set shifting than HCs, these findings were supported in only one study. In general, more robust evidence is needed to confirm alterations in inmates convicted due to violent behavior. These findings highlight the importance of designing and promoting specific cognitive interventions that contribute to the reintegration of inmates into society.

**Systematic review registration:**

https://www.crd.york.ac.uk/prospero/display_record.php?ID=CRD42021252043, identifier CRD42021252043.

## 1. Introduction

Violence is a global public problem of great concern (Bureau of Justice Statistics, 2020). There are currently 6.5 homicides per 100,000 inhabitants in the world (Institute for Economics and Peace [IEP], 2021). A recent report indicate that more than 11 million people are imprisoned awaiting trial or convicted (World Prison Brief, 2020). The growth of the prison population and the high recidivism rates of inmates represent a major social and economic burden on public safety (Navarrete and Austin, 2020). Specifically, since 2000 the inmate population in the world has grown by 24% (Bureau of Justice Statistics, 2020). Recidivism rates of inmates have ranged from 35% to 67% in different countries (Meijers et al., 2015). Deprivation of liberty affects inmates' physical and mental health and increases the development of maladaptive feelings and behaviors that, in the long term, lead to inmate recidivism (Meijers et al., 2017; Wallinius et al., 2019; Cruz et al., 2020).

Neuropsychology has contributed to the understanding of the biological basis of social behavior (Arioli et al., 2020). A meta-analysis (Ogilvie et al., 2011), in which 126 studies involving 14,786 cases were analyzed, found that people with antisocial behaviors presented greater impairments in executive functions than healthy controls (HCs). In this comparison, the groups with more alterations were those with antisocial behaviors, externalizing behavior disorders, and antisocial personality disorders. In this regard, some studies have indicated that people convicted of violent behavior with alterations in executive functions report higher levels of aggression (Wallinius et al., 2019; Alvarado-Grijalba et al., 2020; Cruz et al., 2020) and others suggest that alterations in executive functions are related to social skills, impulsivity, foresight, and judgment (Karlsson et al., 2016; Meijers et al., 2017; Cruz et al., 2020).

Executive functions are cognitive, emotional, and motivational processes essential for regulating behavior, developing adaptive behaviors, and facilitating the social integration of individuals (Ogilvie et al., 2011). A systematic review (Tirapu-Ustárroz et al., 2017), in which 33 studies were explored, indicated that executive functions are composed of cognitive processes that enable control, self-regulation of behavior, processing speed, working memory, verbal fluency, inhibition, dual execution, cognitive flexibility, planning, and decision making. Specifically, there is evidence that alterations in these functions generate cognitive and behavioral manifestations that interfere in the functioning and social adaptation of individuals (Arioli et al., 2020; Cruz et al., 2020).

People with criminal records demonstrate worse performance in planning and monitoring (Seruca and Silva, 2015; Meijers et al., 2017; Delfín et al., 2018), cognitive flexibility (Meijers et al., 2015; Seruca and Silva, 2015; Karlsson et al., 2016; Wallinius et al., 2019), and inhibition (Meijers et al., 2017, 2018; Wallinius et al., 2019). Inmates convicted of violent behavior showed deficits in prefrontal functioning, as well as greater impairments in inhibitory control, set shifting, attention, working memory, planning, and inhibitory responses -the latter being associated with aggressive behaviors- than HCs or inmates convicted of non-violent behavior (Zou et al., 2013; Karlsson et al., 2016; Meijers et al., 2017; Wallinius et al., 2019).

Recent research indicates that inmates convicted of sexual abuse or rape report greater alterations in executive functions than those convicted of non-sexual offenses (Pulido et al., 2017) and non-inmates (Burton et al., 2016; Guamán and Carballo, 2019). This could be because these inmates present executive dysfunctions involving high impulsivity and aggressiveness, as well as difficulty in planning, inhibition, and regulation, the latter being a possible predictor of the frequency in which these individuals commit crimes (Burton et al., 2016; Pulido et al., 2017; Guamán and Carballo, 2019). In addition, this group of inmates reports poorer performance in cognitive flexibility and executive control compared to non-inmates (Burton et al., 2016; Guamán and Carballo, 2019).

A previous review (Meijers et al., 2015) identified that inmates convicted of violent behavior showed greater impairments in inhibition and set shifting than non-inmates. Given the aim of the study and methodological approach, this narrative review used a less restrictive selection of articles, including those with heterogeneous comparison groups (inmates for violent behavior, inmates for other types of behaviors, and HCs), or even without a comparison group. Although this review represents an interesting scientific contribution, the results should be taken with caution due limitations such as the small size of the samples of the included studies and the lack of evaluation of several executive functions in the same population.

For this update, inclusion of new articles was sought using an approach suggested by Meijers et al. (2015). Specifically, this consisted of exploring studies that evaluate groups of inmates for violent behavior in comparison with different controls (e.g., non-violent inmates or HCs). In addition, this made it possible to analyze the extent to which differences in executive function are specific to violent offenders (De Brito et al., 2013; Zou et al., 2013; Chen et al., 2014; Moreno, 2014; Vilà-Balló et al., 2014; Karlsson et al., 2016; Meijers et al., 2017; Sedgwick et al., 2017; Slotboom et al., 2017; Baliousis et al., 2019).

As far as it is known, this is the first study to systematically explore baseline executive functions in inmates convicted of violent behavior compared with inmates convicted of non-violent behavior and HCs. To facilitate the interpretation of the findings, this study also assessed the risk of bias (RoB) of the studies explored. The identification of alterations in executive functions could help to increase the neuropsychological understanding underlying violent behaviors in this population (Santos-Barbosa and Coelho-Monteiro, 2008; Hanlon et al., 2010; Arana et al., 2013; Rocha et al., 2014; Pulido et al., 2017; Carreño et al., 2018; Delfín et al., 2018; Meijers et al., 2018; Cando-Pilatuña et al., 2019; Spenser et al., 2019; Wallinius et al., 2019).

## 2. Materials and methods

### 2.1. Protocol and registration

This systematic review was performed following the Preferred Reporting Items for Systematic Reviews and Meta-analyses (PRISMA; Page et al., 2021). The protocol was registered in Prospective Register of Systematic Reviews (PROSPERO), under registration number CRD42021252043.

### 2.2. Sources of information and search

Systematics searches were performed using PubMed, Scopus, Web of Science, EBSCO, and Embase. The selection of terms was performed considering the Context, How, Issues and Population (CHIP) and search string of the previous review (Meijers et al., 2015). The Boolean search was validated with the support of three external reviewers and a documentalist, using the Peer Review of Electronic Search Strategies (PRESS; McGowana et al., 2016).

The search strategy combined terms related to the population (*prisoner OR incarcerated OR criminal OR violent offenders OR offender*) and executive functions (*executive function OR executive dysfunction OR neurocognitive OR neuropsychological OR neuropsychology*), found in titles, abstracts, and Medical Subject Headings (MeSH). The search strategies employed in each database are detailed in the Supplementary File 1. If possible, the search included the following filters: the period of publication (2007–2023), type of document (article), field (Psychology, Psychiatry, Neuroscience, Health Sciences, and Social Sciences), language (English or Spanish), age ranges (≥18 years), and species (human). The search period was defined considering the results obtained in the review by Meijers et al. (2015). The reference list of included studies was also examined by a reverse citation search for further analysis. The database search was updated until March 6^th^, 2023.

### 2.3. Eligibility criteria

Eligibility criteria were defined based on the Participants, Intervention, Comparison, Outcomes, and Study Design (PICOS; Perestelo-Pérez, 2013). Table 1 details the inclusion and exclusion criteria established in this systematic review. Forensic inmates (such as pedophilic inmates) were excluded from this study since, according to the literature, they are associated with a psychiatric disorder (Berryessa, 2018) that could confound the results of the study. Additionally, juvenile inmates were excluded due to the changes in brain development that occur during this developmental period, which could also confound the findings of this systematic review (Maschi and Dasarathy, 2019).

**Table 1:** Eligibility criteria according to PICOS strategy.

	**Inclusion criteria**	**Exclusion criteria**
[P] Participants	Adult inmates convicted for violent behavior: homicide, attempted homicide, aggravated robbery, armed robbery, physical assault, kidnapping (or attempted), forcible confinement, domestic violence, sexual abuse, or rape (Harris et al., 2002).	Forensic population, juvenile delinquents, or minors.
[I] Intervention	Not applicable.	Not applicable.
[C] Comparison	Adult inmates convicted for violent behavior compared with at least one control group: inmates convicted of nonviolent behavior or HC not deprived of their liberty and with no criminal record.	Other types of comparison groups.
[O] Outcomes	Executive functions: inhibition, cognitive flexibility, working memory, decision making, set shifting, and planning.	Other types of measures.
[S] Study design	Experimental, quasi-experimental, or observational studies in humans; published in peer-reviewed scientific journals; published in English or Spanish; and with a publication date between 2007 and 2023.	Non-original (systematic reviews, meta-analyses, case reports, editorials, guidelines, etc.), dissertations, or qualitative studies.

### 2.4. Data management and study selection

In the first phase, results of the database searches were exported to Mendeley. In the second phase, duplicate articles were automatically detected and removed. In the third phase, titles and abstracts were screened using the Rayyan QCRI Intelligent Systematic Review tool. In the fourth phase, selected full articles were reviewed for compliance with the eligibility criteria. In the fifth phase, data were extracted from the selected full articles in a standardized record format and RoB assessment was performed. All these phases were performed by two independent reviewers (MAC-Q and DQ-M). Discrepancies in the process were agreed with the third (MTC) and fourth reviewer (JPS-M).

### 2.5. Data extraction and coding

The first (MAC-Q) and second (DQ-M) reviewers synthesized all relevant information from the articles in a standardized record format. The data extracted were authors, year of publication, country, study design, mean age, and executive functions evaluated. The statistical parameters analyzed were sample size, means, standard deviations (SDs), significance, and effect size.

### 2.6. Risk of bias (RoB)

RoB was assessed for each eligible study using an adapted version of the Quality Assessment Tool for observational studies from the National Heart, Lung, and Blood Institute (National, Heart, Lung, and Blood Institute [NHLBI], 2018). This risk of bias assessment tool helps reviewers focus on concepts that are key to the internal validity of a study, considering aspects related to study designs and verifying possible bias in methods or research implementation (National, Heart, Lung, and Blood Institute [NHLBI], 2018). The total score of the RoB scale composed of eight items was divided into low (6–8), medium (4–5), and high (≤3). Items were scored 1 point when criteria were met and 0 when criteria were not met, unclear, or could not be determined. The procedure followed for the adaptation of this tool can be found on the Supplementary File 2.

### 2.7. Data analysis

Data were classified according to the sociodemographic characteristics of the sample, outcomes explored, and effect sizes reported. Effect sizes were evaluated according to Cohen's proposal (Albers and Lakens, 2018): small (*d* ≥ 0.2), medium (*d* ≥ 0.5), and large (*d* ≥ 0.8).

## 3. Results

### 3.1. Selection of included studies

**Figure 1:**
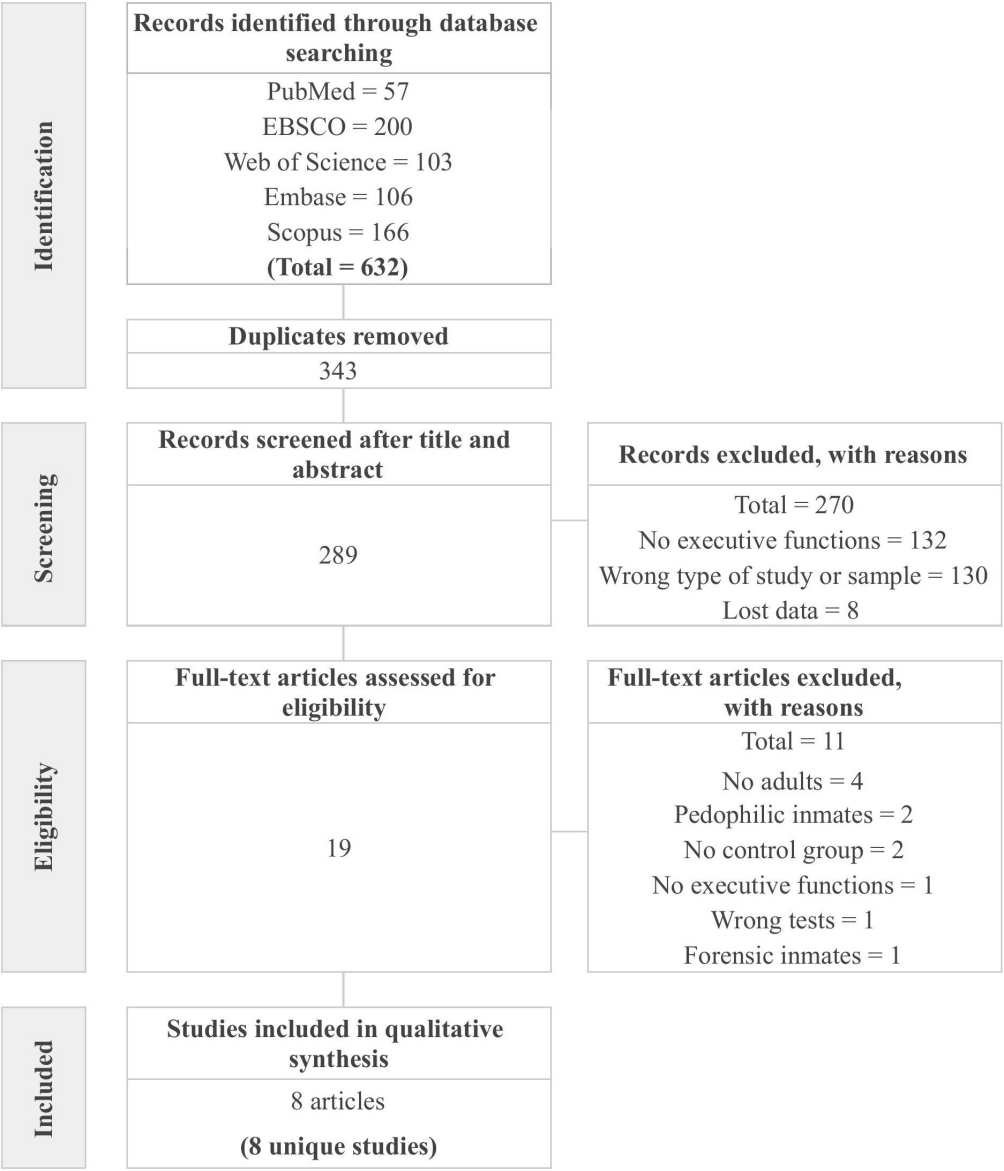
Preferred reporting items for systematic reviews and meta-analyses (PRISMA) flowchart from record identification to study inclusion.

Figure 1 displays the flowchart of the study selection process. The initial search yielded a total of 632 potential studies. In the first phase, after eliminating duplicates, the titles and abstracts of 289 studies were reviewed, of which 19 were selected for full-text review. These studies were excluded because they reported results that were not of interest for this review (*n* = 132); because they did not meet the criteria for population, study design, or types of publications (*n* = 130); and because they were not available (*n* = 8). In the second phase, after reviewing the full texts, 11 studies were eliminated because they included samples with minors (*n* = 4), no control group (*n* = 2), pedophilic inmates (*n* = 2), forensic inmates (*n* = 1), variables measured with non-neuropsychological instruments (*n* = 1), or no variables of interest (*n* = 1). Finally, 8 studies met the eligibility criteria for this systematic review.

### 3.2. Characteristics of the studies

Table 2 presents the characteristics of the included studies. These studies were published between 2007 and 2018. The sample size ranged from 60 to 130 men. The age of the inmates ranged from 18 to 85 years (*M* = 42.13, *SD* = 11.9). In total, four studies (50%) assessed the executive functions of inmates for sexual abuse, with samples ranging from 20 to 68 participants (Becerra-García and Egan, 2014; Becerra-García, 2015; Rodriguez et al., 2017; Rodriguez and Ellis, 2018); and the other four (50%) studies examined inmates convicted of other types of violent crimes (assault, wounding, attempted murder, homicide, manslaughter, and armed robbery, among others), with samples ranging from 20 to 85 participants (Hoaken et al., 2007; Levi et al., 2010; Seruca and Silva, 2016; Meijers et al., 2017). The crimes considered to define an inmate convicted of violent behavior were homicide, attempted homicide, aggravated robbery, armed robbery, physical assault, kidnapping (or attempted), forcible confinement, domestic violence, sexual abuse, or rape. Three studies (Hoaken et al., 2007; Levi et al., 2010; Becerra-García, 2015) used the criteria established by Cornell et al. (1996) and Harris et al. (2002) to define violent behavior. Criteria for classifying nonviolent inmates included crimes such as theft, burglary (nonviolent), drug dealing/possession, or fraud.

**Table 2:** Synthesis of the main characteristics of the studies.

**Authors (year)**	**Country**	**Inmate population sample (mean age)**	**Control population sample (mean age)**	**Executive functions evaluated**
Rodriguez and Ellis (2018)	Australia	11 inmates convicted for the first time for child exploitation material (61 years) and 34 inmates with a history of sexual offenses against children (62 years).	32 inmates for non-sexual offenses (57 years).	Inhibition, decision making, cognitive flexibility, or set shifting.
Rodriguez et al. (2017)	Australia	32 inmates convicted for the first time for sexual offenses (63 years) and 36 inmates convicted for sexual offenses with record (62 years).	32 inmates for non-sexual offenses (55 years).	Inhibition, decision making, cognitive flexibility, or set shifting.
Meijers et al. (2017)	Netherlands	85 inmates for violent behavior (31 years).	45 non-violent inmates (35 years).	Inhibition, planning, working memory, set shifting, and decision making.
Seruca and Silva (2016)	Portugal	13 inmates for violent behavior and 25 non-violent (34 years).	28 healthy control group (36 years).	Inhibition, planning, cognitive flexibility, and working memory.
Becerra-García (2015)	Spain	10 inmates for domestic violence (42 years), 20 for sexual offenses (37 years), and 9 for violent crimes (30 years).	8 non-violent inmates (41 years). 31 healthy control group (38 years).	Inhibition, cognitive flexibility, and set shifting.
Becerra-García and Egan (2014)	Spain	21 incestuous (child molesters) inmates (47 years) and 11 non-incestuous (child molesters) inmates (49.09 years).	28 healthy control group (45 years).	Cognitive flexibility and working memory.
Levi et al. (2010)	Canada	25 predatory inmates (31 years) and 34 irritable inmates (29 years).	30 non-violent inmates (38 years).	Inhibition and decision making.
Hoaken et al. (2007)	Canada	20 inmates for violent behavior (35 years).	20 non-violent inmates (33 years). 20 healthy control group (25 years).	Working memory.

100% of the studies evaluated were cross-sectional.

### 3.3. Risk of bias assessment and effect sizes

As seen in Table 3, 62.5% of the studies obtained a moderate RoB. Effect sizes were reported in 2 out of the 8 studies (Rodriguez et al., 2017; Rodriguez and Ellis, 2018). In the studies where all necessary information was included (Levi et al., 2010; Becerra-García and Egan, 2014; Seruca and Silva, 2016), effect sizes were calculated manually. Due to lack of data, it was not possible to calculate this information in three studies (Hoaken et al., 2007; Becerra-García, 2015; Meijers et al., 2017).

**Table 3:** Risk of bias assessment using an adapted version of the NHLBI.

**Studies**	**Q1**	**Q2**	**Q3**	**Q4**	**Q5**	**Q6**	**Q7**	**Q8**	**Score**	**RoB**
Rodriguez and Ellis (2018)	1	1	1	0	0	1	1	1	6	Low
Rodriguez et al. (2017)	1	1	1	0	0	1	1	1	6	Low
Meijers et al. (2017)	1	1	1	0	0	1	0	1	5	Medium
Seruca and Silva (2016)	1	1	1	0	0	1	0	0	4	Medium
Becerra-García (2015)	1	1	1	1	0	1	1	0	6	Low
Becerra-García and Egan (2014)	1	1	1	0	0	1	1	0	5	Medium
Levi et al. (2010)	1	1	1	0	0	1	1	0	5	Medium
Hoaken et al. (2007)	1	1	1	0	0	1	0	1	5	Medium

Score ranges from 1 to 8. Low (6, 7, 8), medium (4, 5), and high (1, 2, 3) risk of bias. Q1. Was the research question or objective in this paper clearly stated? Q2. Was the study population clearly specified and defined? Q3. Were all the subjects selected or recruited from the same or similar populations (including the same period)? Were inclusion and exclusion criteria for being in the study prespecified and applied uniformly to all participants? Q4. Was a sample size justification, power description, or variance and effect estimates provided? Q5. For exposures that can vary in amount or level, did the study examine different levels of the exposure as related to the outcome (e.g., categories of exposure, or exposure measured as continuous variable)? Q6. Were the outcome measures (dependent variables) clearly defined, valid, reliable, and implemented consistently across all study participants? Q7. Were key potential confounding variables measured and adjusted statistically for their impact on the outcome(s)? Q8. The proportion of participants with missing data in the variable is irrelevant (or is adequately justified to be irrelevant) or it is justified that statistical techniques to deal with missing data are appropriate (e.g., weighting adjustments or imputation methods).

### 3.4. Evidence on executive functions

The executive functions measured across these eight studies were: inhibition (*n* = 6), cognitive flexibility (*n* = 5), working memory (*n* = 4), decision making (*n* = 4), set shifting (*n* = 2), and planning (*n* = 2). Table 4 shows the scoring in each task.

**Table 4:** Scoring in each task.

**Study**	**Measures/tasks**	**Executive function**	**Scoring**	
			**Higher score**	**Lower score**
Rodriguez and Ellis (2018)	Inhibition	Hayling test	Better performance	Poor performance
		Controlled oral word association test	Better performance	Poor performance
	Decision making	Iowa gambling task	Better performance	Poor performance
	Cognitive flexibility	Trail making test (score)	Better performance	Poor performance
		Trail making test (time)	Poor performance	Better performance
Rodriguez et al. (2017)	Inhibition	Hayling test	Better performance	Poor performance
		Controlled oral word association test	Better performance	Poor performance
	Decision making	Iowa gambling task	Better performance	Poor performance
	Cognitive flexibility	Trail making test (score)	Better performance	Poor performance
		Trail making test (time)	Poor performance	Better performance
Meijers et al. (2017)	Inhibition	Stop-signal task (reaction time)	Poor performance	Better performance
	Planning	Stockings of Cambridge	Better performance	Poor performance
	Working memory	Spatial working memory task	Poor performance	Better performance
	Set shifting	Intra-extra dimensional set-shift task	Poor performance	Better performance
	Decision making	Cambridge gambling task	Better performance	Poor performance
Seruca and Silva (2016)	Planning	Porteus mazes test (TA)	Better performance	Poor performance
	Inhibition	Porteus mazes test (*Q* score)	Poor performance	Better performance
		Stroop color and word test	Better performance	Poor performance
	Cognitive flexibility	Trail making test (score)	Better performance	Poor performance
		Trail making test (time)	Poor performance	Better performance
	Working memory	Digit span backwards	Better performance	Poor performance
Becerra-García (2015)	Cognitive flexibility, inhibition, and set shifting	Trail making test (score)	Better performance	Poor performance
		Trail making test (time)	Poor performance	Better performance
Becerra-García and Egan (2014)	Cognitive flexibility	Trail making test (score)	Better performance	Poor performance
		Trail making test (time)	Poor performance	Better performance
	Working memory	Digit span	Better performance	Poor performance
Levi et al. (2010)	Inhibition	Integrated visual and auditory continuous performance test	Better performance	Poor performance
	Decision making	Iowa gambling task	Better performance	Poor performance
Hoaken et al. (2007)	Working memory	Conditioned non-spatial association test	Poor performance	Better performance
		Concrete subject-ordered working memory test	Poor performance	Better performance
		Abstract subject-ordered working memory test	Poor performance	Better performance

#### 3.4.1. Inhibition (six studies)

Seruca and Silva (2016) reported that inmates convicted of violent behavior (*n* = 13; *M* = 60.15, *SD* = 36.42) presented significantly poorer performance on the Porteus Mazes Test *Q* score than the control group (*n* = 28, *M* = 32.57, *SD* = 20.42; *p* = 0.01), with a large effect size (*d* = 0.99). In the Stroop test, no significant differences were found between inmates for violent behavior and the control group (*p* = 0.73), as well as between inmates in general (including non-violent inmates) and the control group (*p* = 0.95).

Meijers et al. (2017) found that inmates convicted of violent behavior (*n* = 85) performed significantly worse on the Stop-Signal Task test than those convicted of non-violent behavior (*n* = 45, *p* = 0.02). Levi et al. (2010) indicated that irritable inmates convicted of violent behavior (emotional violent crimes) performed significantly more poorly (*n* = 34, *M* = 81.41, *SD* = 18.26) on the full impulsivity scale on The Integrated Visual and Auditory Continuous Performance Test than predatory inmates (premeditated non-emotional violent crimes; *n* = 25, *M* = 95.08, *SD* = 17.17, *p* = 0.001, *d* = −0.77) and those convicted of non-violent behavior (*n* = 30, *M* = 96.40, *SD* = 15.29, *p* = 0.001, *d* = −0.89).

Rodriguez et al. (2017) and Rodriguez and Ellis (2018) found significant differences in inmates convicted of sex offenses on the Hayling test. The Rodriguez et al. (2017) study identified that both first-time sex offender inmates (*n* = 32; *M* = 3.1, *SD* = 1.6) and inmates with a record of sexual offenses (*n* = 36; *M* = 3.5, *SD* = 1.9) had significantly higher dysfunction (*p* < 0.001, in both cases) on the total scale of the Hayling test than inmates convicted of non-sexual offenses (*n* = 32; *M* = 5.2, *SD* = 1.5), with a large effect size (*d* = 1.4 and 1.0, respectively). However, in this same study, there were no significant differences between the sex offense groups. In the Rodriguez and Ellis (2018) study, inmates with a history of sexual offenses (*n* = 34; *M* = 3.6, *SD* = 1.9) presented significantly poorer performance on this same test (*p* = 0.002; *d* = 0.9) than inmates for non-sexual offenses (*n* = 32; *M* = 5.2, *SD* = 1.5). In addition, no statistically significant differences were displayed between sex offense and non-sex offense inmates.

Regarding the COWAT test, the study by Rodriguez et al. (2017) evidenced that inmates convicted for the first time of sexual offenses (*M* = 31.9, *SD* = 13.2) presented significantly worse performance on inhibitory responding (*p* < 0.05; *d* = 0.43) than inmates with a history (*M* = 37.4, *SD* = 12.3) and non-sexual inmates (*M* = 43.9, *SD* = 10.5; *p* < 0.001; *d* = 1.00). Despite using the same test, no significant results between comparison groups were presented in the Rodriguez and Ellis (2018) study.

Becerra-García (2015) compared 10 domestic violence inmates, 20 sex offense inmates, 9 violent crime inmates, 8 non-violent inmates, and 31 control group inmates using the Trail Making Test. This study identified that the sex crime and domestic violence groups performed significantly poorer on inhibitory ability than the control group (*p* = 0.001). In contrast, non-violent inmates did not show significant differences compared to the control group.

#### 3.4.2. Cognitive flexibility (five studies)

Seruca and Silva (2016) found that the general inmate group (*n* = 38) performed significantly worse on the Trail Making Test than the control group (*n* = 28, *p* = 0.008; *d* = 0.61). Becerra-García (2015) found that on the Trail Making Test the domestic violence and sexual offenses groups (*n* = 30) performed significantly worse on the *post-hoc* tests than the control group (*n* = 31, *p* = 0.001). Rodriguez et al. (2017) identified that there were no statistically significant differences on the Trail Making Test-B between the first-time sexual violence group (*n* = 32, *M* = 111.7, *SD* = 59.2) and the repeat sexual violence group (*n* = 36, *M* = 124.8, *SD* = 45.9), although both sexual offender groups performed significantly worse on this task than non-sexual inmates (*n* = 32; *M* = 86.8, *SD* = 28.5; *p* < 0.05, in both cases; *d* = 0.6 and *d* = 1.00, respectively).

Even though in the Rodriguez and Ellis (2018) study both the group of inmates first convicted of sexual offenses (*n* = 11, *M* = 38.8, *SD* = 12.3) and group with history (*n* = 34, *M* = 37.7, *SD* = 12.6) presented poorer performance on the FAS-COWAT test than non-sexual inmates (*n* = 32, *M* = 43.9, *SD* = 10.5), no significant differences were found between the two sexual offender groups. Becerra-García and Egan (2014) indicated that incestuous inmates (*n* = 21, *M* = 155.14, *SD* = 70.24) and non-incestuous inmates (*n* = 11, *M* = 145.09, *SD* = 68.71) presented inferior performance on all tasks of the Trail Making Test than the control group (*n* = 28, *M* = 70.04, *SD* = 26.66, *p* < 0.01), with large effect sizes (*d* = 1.60 and 1.44, respectively). However, there were no significant differences between inmate groups.

#### 3.4.3. Working memory (four studies)

Seruca and Silva (2016) evidenced no statistically significant differences in the Digit Span-Backwards test between inmates (*n* = 38, *M* = 5.97, *SD* = 1.71) and the control group (n = 28, *M* = 6.25, *SD* = 1.53; *p* = 0.39). Meijers et al. (2017) also found no significant differences on the Spatial Working Memory Task test between violent inmates (*n* = 85) and non-violent inmates (*n* = 45). In contrast, Becerra-García and Egan (2014) detected significantly lower scores on section DF of the Digit Span test in incestuous inmates (*n* = 21, *M* = 6.4, *SD* = 1.56) than the control group (*n* = 28, *M* = 9.39, *SD* = 1.95; *p* < 0.01; *d* = −1.67). Similarly, incestuous inmates (*M* = 4.05, *SD* = 1.49) demonstrated poorer performance on the DB section of the *post-hoc* than non-incestuous (*n* = 11, *M* = 5.82, *SD* = 1.83; *p* = 0.02; *d* = −1.06) and the control group (*M* = 6.46; *SD* = 1.85). Non-incestuous inmates did not show significant differences compared to the control group.

Hoaken et al. (2007) transformed executive performance measures (Conditioned Non-Spatial-Association Test, Concrete Subject-Ordered Working Memory Test and Abstract Subject-Ordered Working Memory Test) to construct a single variable measuring working memory. In this study, no statistically significant differences were found between violent (*n* = 20) and non-violent (*n* = 20) behavior inmates, but significantly poorer performance in these two groups compared to the control group (*n* = 20, *p* < 0.01) was observed.

#### 3.4.4. Decision making (four studies)

Levi et al. (2010) found significantly superior performance on the Iowa Gambling Test (IGT) in non-violent inmates (*n* = 30, *M* = 9.23, *SD* = 7.90) than in irritable (*n* = 34, *M* = 2.53, *SD* = 10.08; *p* = 0.009; *d* = 0.73) and predatory (*n* = 25, *M* = 3.68, *SD* = 7.93; *p* = 0.057; *d* = 0.70) inmates. Although no significant differences were present between the two violent groups, irritable inmates performed worse than non-violent inmates. Rodriguez et al. (2017) and Rodriguez and Ellis (2018) used the IGT for the assessment of decision-making and Meijers et al. (2017) the Cambridge Gambling Task; however, in none of these three studies were significant differences found between their comparison groups.

#### 3.4.5. Set shifting (two studies)

Meijers et al. (2017) found no statistically significant differences in performance on the Intra-Extra Dimensional Set-Shift Task test between inmates for violent behavior (*n* = 85) and inmates for non-violent behavior (*n* = 45). In contrast, Becerra-García (2015) found significantly worse performance on the Trail Making Test between the domestic violence inmate groups (*n* = 10) and the control group (*n* = 31, *p* = 0.004); and between the sexual behavior inmates (*n* = 20) and the control group (*p* = 0.004).

#### 3.4.6. Planning (two studies)

Seruca and Silva (2016) indicated significantly poorer performance on the Porteus Maze Task in inmates for violent behavior (*n* = 13, *M* = 15.57, *SD* = 2.277) than in the control group (*n* = 28, *M* = 18, *SD* = 2.09; *p* = 0.040; *d* = 11.18). In contrast, Meijers et al. (2017) evidenced no statistically significant differences in performance on the Stockings of Cambridge test between their study groups.

### 3.5. Alterations in executive functions

Tables 5, 6 compares the evidence identified between inmates convicted of violent behavior and inmates convicted of non-violent behavior or HCs, respectively.

**Table 5:** Synthesis of the identified evidence of VI vs. NV.

**Executive functions**	**Studies (*n*)**	**VI (*N*)**	**NV (*N*)**	**Directionality VI *vs*. NV (*n*, %)**	**Significant differences VI *vs*. NV (*n*, %)**
Inhibition	4	257	139	↓ Lower levels (4/4, 100%)	(4/4, 100%)
Cognitive flexibility	4	165	97	= Equal levels (3/4, 75%) ↓ Lower levels (1/4, 25%)	(1/4, 25%)
Working memory	3	118	90	= Equal levels (3/3, 100%)	(0/3, 0%)
Decision making	4	257	139	= Equal levels (3/4, 75%) ↓ Lower levels (1/4, 25%)	(1/4, 25%)
Set shifting	1	85	45	= Equal levels (1/1, 100%)	(0/1, 0%)
Planning	2	98	70	= Equal levels (2/2, 100%)	(0/2, 0%)

Significant differences were determined using p ≤ 0.05. VI, inmates convicted of violent behavior; NV, inmates convicted of nonviolent behavior; N, number of total individuals; n, number of studies. The study by Becerra-García and Egan (2014) was not included in this comparison, as it did not include a control group of inmates convicted of non-violent behavior.

**Table 6:** Synthesis of the identified evidence of VI vs HC.

**Executive functions**	**Studies (*n*)**	**VI (*N*)**	**HC (*N*)**	**Directionality VI *vs*. HC (*n*, %)**	**Significant differences VI *vs*. HC (*n*, %)**
Inhibition	2	52	59	↓ Lower levels (2/2, 100%)	(2/2, 100%)
Cognitive flexibility	3	84	87	= Equal levels (1/3, 33.3%) ↓ Lower levels (2/3, 66.6%)	(2/3, 66.6%)
Working memory	3	65	76	= Equal levels (1/3, 33.3%) ↓ Lower levels (2/3, 66.6%)	(2/3, 66.6%)
Set shifting	1	39	31	↓ Lower levels (1/1, 100%)	(1/1, 100%)
Planning	1	13	28	↓ Lower levels (1/1, 100%)	(1/1, 100%)

Significant differences were determined using p ≤ 0.05. VI, inmates convicted of violent behavior; NV, inmates convicted of nonviolent behavior; HC, healthy control group (i.e., individuals with no criminal record). N, number of total individuals; n, number of studies.

## 4. Discussion

Inmates have historically been a stigmatized, neglected, and underserved population in need of interventions that promote their wellbeing and reintegration into society. This systematic review provided up-to-date evidence, with a considerable temporal range of studies (2007 to 2018) including comparisons with other groups (i.e., inmates convicted of non-violent behavior and HCs) on executive function alterations in inmates convicted of violent behavior. Consistent with previous research, it was identified that, in general, inmates convicted of violent behavior displayed more alterations in executive functions than inmates convicted of non-violent behavior, and non-inmates (De Brito et al., 2013; Zou et al., 2013; Chen et al., 2014; Vilà-Balló et al., 2014; Karlsson et al., 2016; Meijers et al., 2017; Sedgwick et al., 2017; Slotboom et al., 2017; Baliousis et al., 2019). Similarly, inmates convicted exclusively of sexual offenses showed more alterations in executive functions than inmates convicted of non-sexual offenses or HCs (Burton et al., 2016; Guamán and Carballo, 2019).

These findings indicated that inmates convicted of violent behavior presented greater impairments in inhibition (Levi et al., 2010; Becerra-García, 2015; Seruca and Silva, 2016; Meijers et al., 2017; Rodriguez et al., 2017; Rodriguez and Ellis, 2018) than inmates convicted of non-violent behavior (Levi et al., 2010; Becerra-García, 2015; Seruca and Silva, 2016; Meijers et al., 2017; Rodriguez et al., 2017; Rodriguez and Ellis, 2018) and HCs (Becerra-García, 2015; Seruca and Silva, 2016). Four out of the six studies that evaluated this function (Levi et al., 2010; Becerra-García, 2015; Seruca and Silva, 2016; Meijers et al., 2017; Rodriguez et al., 2017; Rodriguez and Ellis, 2018) reported large (Levi et al., 2010; Seruca and Silva, 2016; Rodriguez and Ellis, 2018) or moderate (Rodriguez et al., 2017) effect sizes. In addition, these studies obtained a low to moderate risk of bias.

The systematic review by Meijers et al. (2015) reported inconsistent evidence on alterations in inhibition (Munro et al., 2007; Schiffer and Vonlaufen, 2011) in inmates convicted of violent behavior and inmates convicted of non-violent behavior. This inconsistency could be since the two studies that explored this function included inmates convicted of different types of violent offenses (instrumental in one study and reactive in another). In the current systematic review, in contrast, the evidence on inhibition analyzed were consistent (Levi et al., 2010; Becerra-García, 2015; Seruca and Silva, 2016; Meijers et al., 2017; Rodriguez et al., 2017; Rodriguez and Ellis, 2018). Similarly, greater alterations in inhibition were identified in inmates convicted of violent behavior than in inmates convicted of nonviolent behavior (Levi et al., 2010; Meijers et al., 2017; Rodriguez et al., 2017; Rodriguez and Ellis, 2018) and HCs (Becerra-García, 2015; Seruca and Silva, 2016).

As several studies have pointed out, alterations in inhibition could explain the difficulty of inmates to control their impulses (Levi et al., 2010; Becerra-García, 2015; Seruca and Silva, 2016; Meijers et al., 2017; Rodriguez et al., 2017; Rodriguez and Ellis, 2018), as well as predict recidivism and the emission of aggressive behaviors (Zou et al., 2013; Karlsson et al., 2016; Wallinius et al., 2019). It is important to intervene in inhibitory control in inmates because this executive function allows them to regulate impulsive or automatic responses that may be harmful to themselves or others (Becerra-García, 2015). Inhibitory control also promotes cognitive flexibility, working memory, and emotional regulation (Seruca and Silva, 2016; Rodriguez et al., 2017), which are necessary skills for environmental adaptation and social reintegration. By improving inhibitory control, inmates can act in a specific and socially appropriate manner when faced with different situations (Becerra-García, 2015). In this sense, the assessment of inhibition impairment could be critical for predicting criminal or violent behavior in new and recidivist inmates.

None of the studies included in this systematic review found statistically significant alterations in working memory (Hoaken et al., 2007; Seruca and Silva, 2016; Meijers et al., 2017), set shifting (Meijers et al., 2017), and planning (Seruca and Silva, 2016; Meijers et al., 2017) in inmates convicted of violent behavior compared to those convicted of nonviolent behavior. Statistically significant differences in cognitive flexibility (Seruca and Silva, 2016; Rodriguez et al., 2017; Rodriguez and Ellis, 2018) and decision-making (Meijers et al., 2017; Rodriguez et al., 2017; Rodriguez and Ellis, 2018) were also not identified in three out of four studies.

In two out of the three studies explored, inmates convicte d of violent behavior presented greater alterations in cognitive flexibility (Becerra-García and Egan, 2014; Becerra-García, 2015; Seruca and Silva, 2016) and working memory (Hoaken et al., 2007; Becerra-García and Egan, 2014) than HCs. Although with evidence supported by the results of a single small-sample study, greater alterations in planning (Seruca and Silva, 2016) and set shifting (Becerra-García, 2015) were identified in inmates convicted of violent behavior than in HCs. Nevertheless, these studies obtained a low to moderate risk of bias.

Improvements in cognitive flexibility, working memory, and planning may have a significant impact on reducing violent behavior among inmates (Vadini et al., 2018; Molleman et al., 2022; Romero-Martínez et al., 2022). Specifically, enhancing these executive functions could help prisoners regulate their emotions, inhibit impulsive behavior, and plan and execute non-violent problem-solving strategies (Seruca and Silva, 2016; Valizadeh et al., 2020). As a result, prisoners may be less likely to engage in violent behavior, both within prison and after their release (Meijers et al., 2018; Molleman et al., 2022). Moreover, improved cognitive functioning can lead to better decision-making skills and increased self-control, which are crucial for successful reintegration into society.

In the present systematic review, half of the articles included evaluated several executive functions at the same time, which allowed for a greater exploration of the results of each sample. In addition, these studies showed the common characteristic of being recently published. In particular, the need to explore a larger set of executive functions in each study was noted within the recommendations of the previous review (Meijers et al., 2015). In accordance with the above, it can be observed that not only has research on this topic continued, but it has been gradually developing, improving its methodologies, and broadening the spectrum of assessment of executive functioning (Richter, 2010; Larrota et al., 2018). Although the tendency of the articles to explore a greater number of executive functions seems evident, the possible role of confounding variables has been little explored in the studies included in this systematic review.

Confounding is a systematic error that occurs frequently in observational studies (Viswanathan et al., 2013). The distortion introduced by an unexplored confounding factor can be a fundamental aspect for the proper interpretation of an effect (Arah, 2017). In this sense, it is important to explore the role of confounding variables in studies included in systematic reviews considering they can affect the validity and precision of estimates of the effect of an exposure on an outcome (Metelli and Chaimani, 2020). If not adequately controlled, they can introduce bias into the analysis and lead to erroneous conclusions (Arah, 2017; Metelli and Chaimani, 2020). Therefore, the risks of bias and confounding that may be present in the studies included in the review should continue to be considered (Viswanathan et al., 2013).

Specifically, exploring the possible confounding role of sociodemographic and clinical characteristics (i.e., sex, age, education level, occupation, socioeconomic status, marital status, place of residence, diagnosis of mental disorders, psychoactive substance use, frontal circuit maturity, type of cognitive task, degree of development of executive function, diagnoses of mental disorders, head injury or history of stroke) in the comparison of executive functions between the inmate population and control groups (Álvis et al., 2014; Mercado et al., 2014; Arana-Medina et al., 2019) is an aspect of great relevance for future lines of research.

### 4.1. Study limitations and strengths

The findings of this systematic review should be analyzed considering the following limitations: (a) differences in conceptualizations of executive functions; (b) variety of instruments used to measure executive functions; (c) small sample sizes; (d) limited quantity of evidence for each outcome; (e) moderate risk of bias of the included studies, which could imply inaccuracies in the conclusions; and (f) only cross-sectional studies were assess. In contrast, the strengths of this review were: (a) expanding the inclusion criterion to include studies with non-violent inmates and/or HCs as a control group; (b) updating of the evidence by including studies until 2023, a range that had not been explored previously; (c) inclusion of studies that assessed more than one executive function, which facilitates a more accurate exploration of the findings for each sample; and (d) inclusion of the quality assessment tool to estimate the RoB.

### 4.2. Reflections and recommendations for future research

This study provided a broader picture of the development of research on executive functions in the inmate population. It also highlights the need for further research on executive functions beyond neuropsychological assessments. Expanding knowledge about this phenomenon and conducting targeted interventions in this population could have a direct or indirect impact on crime and recidivism rates. This is especially critical in populations that have been poorly studied, where the knowledge gap about executive functions is unquestionable, such as inmates in American countries, whose rates of crime and recidivism continue to increase. This is an aspect that has overflowed the capacity of the prison and penitentiary system.

The failure of prison as a resocialization system and recidivism potentiates a dangerous spiral. The problems attributed to crime encourages legal initiatives that, instead of decreasing criminality, strengthen the identification of inmates with the criminal environment, promotes recidivism, and generates more skilled criminals. This makes it easier not to differentiate between degrees of dangerousness of inmates, nor psychological or social circumstances. Identifying them with a subculture of violence and illegality that apparently does not present ways to overcome. This systematic review serves as an input to consider the need for prevention programs, as well as the importance of designing and promoting specific cognitive interventions rather than general executive function interventions for inmates. Stronger evidence is needed to confirm the magnitude of alterations in other executive function processes in inmates convicted of violent behavior to understand the role of executive functions in violent behavior.

In the future, longitudinal studies with representative samples comparing groups of inmates convicted for different types of crimes are suggested. This will allow to deepen the knowledge about the phenomenon of executive function, the possible differences between inmate populations, and the development of a differential profile according to the crime. This research could contribute to develop of interdisciplinary programs for the prevention of executive dysfunctions, as well as for the promotion and intervention in the executive function of inmates, which are so necessary for reintegration into society and to contemplate establishing public policies to guide the processes of change in this situation. Finally, considering the scientific interest generated by the exploration of executive functions in this population in recent years, systematic reviews are suggested to be updated when more evidence is published.

## 5. Conclusions

This systematic review presented evidence of impaired executive functioning in inmates sentenced for violent behavior compared with inmates sentenced for non-violent behavior or HCs. Although previous studies found differences in executive functions such as inhibition, cognitive flexibility, working memory, and planning, among others, compared to heterogeneous groups (i.e., inmates for violent behavior, inmates for other types of behavior, and/or HC), the current systematic review suggests that inmates convicted of violent behaviors compared exclusively with inmates convicted of non-violent behaviors and healthy controls (HCs) had greater deficiencies in inhibition, which is responsible for stopping, controlling, and modifying impulsive behaviors and executing more socially appropriate behaviors. These findings reaffirms the importance of focusing on inhibition, as well as continuing research on other specific cognitive processes (e.g., cognitive flexibility, working memory, set shifting, and planning, among others), in neuropsychological interventions.

## Data availability statement

The original contributions presented in the study are included in the article/Supplementary material, further inquiries can be directed to the corresponding authors.

## Author contributions

MC-Q and DQ-M conceived the idea for this article, conducted the search for studies, evaluated the articles, and drafted the manuscript, tables, and figure. MC and JS-M resolved discrepancies, guided, reviewed, and proofread the entire process. All authors contributed to the article and approved the submitted version.

## References

[B1] Albers C. Lakens D. (2018). When power analyses based on pilot data are biased: inaccurate effect size estimators and follow-up bias. J. Exp. Soc. Psychol. 74, 187–195. 10.1016/j.jesp.2017.09.004

[B2] Alvarado-Grijalba S. Pulido-Suárez C. Rincón-Lozada C. (2020). Desempeño de la función ejecutiva por áreas, en internos condenados por homicidio involuntario y doloso. Arch. Neurosci. 25, 19–31. 10.31157/an.v25i1.192

[B3] Álvis A. Arana M. Restrepo J. C. (2014). Propuesta de rehabilitación neuropsicológica de la atención, las funciones ejecutivas y empatía en personas con diagnóstico de trastorno antisocial de la personalidad, desvinculadas del conflicto armado colombiano. Rev. Virt. Univer. Católica del Norte 42, 138–153.

[B4] Arah O. A. (2017). Bias analysis for uncontrolled confounding in the health sciences. Annu. Rev. Public Health 38, 23–38. 10.1146/annurev-publhealth-032315-021644 28125388

[B5] Arana C. Gómez M. Molina D. (2013). Alteración de las funciones ejecutivas en personas vinculadas al conflicto armado colombiano. Katharsis 15, 133–151.

[B6] Arana-Medina C. M. Álvis-Rizzo A. Restrepo-Botero J. C. Hoyos-Zuluaga E. (2019). Rehabilitación de las funciones ejecutivas y la cognición social, en sujetos con trastorno de personalidad antisocial, vinculadas al conflicto armado en Colombia. Revista Argentina de Clínica Psicológica. 28, 92–104. 10.24205/03276716.2018.1073

[B7] Arioli M. Ricciardi E. Cattaneo Z. (2020). Social cognition in the blind brain: a coordinate-based meta-analysis. Hum. Brain Map. 42, 1243–1256. 10.1002/hbm.25289 33320395 PMC7927293

[B8] Baliousis M. Duggan C. McCarthy L. Huband N. Völlm B. (2019). Executive function, attention, and memory deficits in antisocial personality disorder and psychopathy. Psych. Res. 278, 151–161. 10.1016/j.psychres.2019.05.046 31200194

[B9] Becerra-García J. A. (2015). Neuropsychology of domestic violence: a comparative preliminary study of executive functioning. Med. Sci. Law 55, 35–39. 10.1177/0025802414525148 24644222

[B10] Becerra-García J. A. Egan V. (2014). Neurocognitive functioning and subtypes of child molesters: poorer working memory differentiates incestuous from non-incestuous offenders. Psych. Psychol. Law 21, 585–590. 10.1080/13218719.2013.873974

[B11] Berryessa C. M. (2018). The effects of psychiatric and “biological” labels on lay sentencing and punishment decisions. J. Exp. Criminol. 14, 241–256. 10.1007/s11292-018-9322-x

[B12] Bureau of Justice Statistics. (2020). Recidivism. Available online at: https://www.bjs.gov/index.cfm?ty=tp&tid=17

[B13] Burton D. Demuynck S. Yoder J. R. (2016). Executive dysfunction predicts delinquency but not characteristics of sexual aggression among adolescent sexual offenders. Sex. Abuse J. Res. Treatment 28, 707–721. 10.1177/1079063214556357 25428928

[B14] Cando-Pilatuña R. I. Guamán-Caba V. N. Carballo-Briones K. E. (2019). Funciones ejecutivas en personas privadas de libertad. Centro de Rehabilitación Social Guaranda, 2018-2019 (Degree dissertation). Universidad Nacional, de Chimborazo, Ecuador. Available online at: http://dspace.unach.edu.ec/handle/51000/5737

[B15] Carreño M. F. Jiménez A. M. Rincón C. F. (2018). Evaluación del desempeño neuropsicológico en condenados por homicidio simple y homicidio agravado. Archivos de Neurociencias. 22, 50–63.

[B16] Chen C. Y. Muggleton N. G. Chang J. R. (2014). Inefficiency of post-error adjustment in impulsive violent offenders. Neuroreport 25, 1024–1029. 10.1097/WNR.0000000000000212 25026532

[B17] Cornell D. G. Warren J. Hawk G. Stafford E. Oram G. Pine D. (1996). Psychopathy in instrumental and reactive violent offenders. J. Consult. Clin. Psychol. 64, 783–790. 10.1037/0022-006X.64.4.783 8803369

[B18] Cruz A. R. Castro-Rodrigues A. Barbosa F. (2020). Executive dysfunction, violence and aggression. Aggress. Viol. Behav. 51, 101380. 10.1016/j.avb.2020.101380

[B19] De Brito S. A. Viding E. Kumari V. Blackwood N. Hodgins S. (2013). Cool and hot executive function impairments in violent offenders with antisocial personality disorder with and without psychopathy. PLoS ONE 8, e65566. 10.1371/journal.pone.0065566 23840340 PMC3688734

[B20] Delfín C. Andiné P. Hofvander B. Billstedt E. Wallinius M. (2018). Examining associations between psychopathic traits and executive functions in incarcerated violent offenders. Front. Psych. 9, 310. 10.3389/fpsyt.2018.00310 30050476 PMC6050384

[B21] Guamán N. Carballo K. (2019). Funciones ejecutivas en personas privadas de libertad. Centro de rehabilitación social Guaranda, 2018–2019 (Degree dissertation). Universidad Nacional de Chimborazo, Ecuador. Available online at: https://1library.co/document/qmj50k8q-funciones-ejecutivas-personas-privadas-libertad-centro-rehabilitacion-guaranda

[B22] Hanlon R. E. Rubin L. H. Jensen M. Daoust S. (2010). Neuropsychological features of indigent murder defendants and death row inmates in relation to homicidal aspects of their crimes. Arch. Clin. Neuropsychol. 25, 1–13. 10.1093/arclin/acp099 20015966

[B23] Harris G. T. Rice M. E. Cormier C. A. (2002). Prospective replication of the violence risk appraisal guide in predicting violent recidivism among forensic patients. Law Hum. Behav. 26, 377–394. 10.1023/A:1016347320889 12182529

[B24] Hoaken P. Allaby D. Earle J. (2007). Executive cognitive functioning and the recognition of facial expressions of emotion in incarcerated violent offenders, non-violent offenders, and controls. Aggress. Behav. 33, 412–421. 10.1002/ab.20194 17683105

[B25] Institute for Economics Peace [IEP]. (2021). Global Peace Index 2021: Measuring Peace in a Complex World. Available online at: https://www.visionofhumanity.org/wp-content/uploads/2021/06/GPI-2021-web-1.pdf (accessed October, 2022).

[B26] Karlsson L. Soveri A. Saarinen M. Weizmann-Henelius G. (2016). The role of set-shifting ability in differentiating between subgroups of female violent offenders. J. Foren. Psych. Psychol. 27, 745–759. 10.1080/14789949.2016.1152589

[B27] Larrota R. Gaviria A. M. Mora C. Arenas A. (2018). Aspectos criminogénicos de la reincidencia y su problema. Revista de la Universidad Industrial de Santander Salud 50, 158–165. 10.18273/revsal.v50n2-2018007

[B28] Levi M. Nussbaum D. Rich J. (2010). Neuropsychological and personality characteristics of predatory, irritable, and non-violent offenders. Crim. Just. Behav. 37, 633–655. 10.1177/0093854810362342

[B29] Maschi T. Dasarathy D. (2019). Aging with mental disorders in the criminal justice system: a content analysis of the empirical literature. Int. J. Offend. Therapy Compar. Criminol. 63, 2103–2137. 10.1177/0306624X19843885 31068046

[B30] McGowana J. Sampsonc M. Salzwedeld D. Cogoe E. Foersterf V. Lefebvre C. (2016). PRESS peer review of electronic search strategies: 2015. J. Clin. Epidemiol. 75, 40–46. 10.1016/j.jclinepi.2016.01.021 27005575

[B31] Meijers J. Harte J. Meynen G. Cuijpers P. (2017). Differences in executive functioning between violent and non-violent offenders. Psychol. Med. 47, 1784–1793. 10.1017/S0033291717000241 28173890

[B32] Meijers J. Harte J. M. Jonker F. A. Meynen G. (2015). Prison brain? Executive dysfunction in prisoners. Front. Psychol. 6, 1–6. 10.3389/fpsyg.2015.00043 25688221 PMC4311616

[B33] Meijers J. Harte J. M. Meynen G. Cuijpers P. Scherder E. J. (2018). Reduced self-control after 3 months of imprisonment: a pilot study. Front. Psychol. 9, 1–7. 10.3389/fpsyg.2018.00069 29449824 PMC5799890

[B34] Mercado C. Arango G. Segura S. (2014). Cien años de construcción de un sistema carcelario y penitenciario en Colombia. Bogotá, DC: Instituto Nacional Penitenciario y Carcelario. Available online at: https://www.inpec.gov.co/documents/20143/64716/RESE%C3%91A+HISTORICA+DOCUMENTAL+100+A%C3%91OS+PRISIONES.pdf/dd03098c-a95e-4f35-50cf-ac703a1573af (accessed October, 2022).

[B35] Metelli S. Chaimani A. (2020). Challenges in meta-analyses with observational studies. BMJ Ment Health 23, 83–87. 10.1136/ebmental-2019-300129 32139442 PMC10231593

[B36] Molleman P. W. Molleman L. Schilder C. Bulten B. H. Brazil I. A. (2022). Can measures of cognitive flexibility and inhibition distinguish forensic psychiatric inpatients from prisoners? J. Foren. Psych. Psychol. 33, 371–388. 10.1080/14789949.2022.2070523

[B37] Moreno M. (2014). Estudio comparativo del perfil neuropsicológico prefrontal entre sujetos con conductas psicopáticas y/o delictivas y sujetos normales, en el context del peritaje forense. Revista Facultad de Ciencias Médicas (Quito). 39, 42–52.

[B38] Munro G. E. Dywan J. Harris G. T. McKee S. Unsal A. Segalowitz S. J. (2007). Response inhibition in psychopathy: the frontal N2 and P3. Neurosci. Lett. 418, 149–153. 10.1016/j.neulet.2007.03.017 17418489

[B39] National Heart Lung, Blood Institute [NHLBI].. (2018). Study Quality Assessment Tools. Bethesda: National Heart, Lung, and Blood Institute. Available online at: https://www.nhlbi.nih.gov/health-topics/study-quality-assessment-tools (accessed October, 2022).

[B40] Navarrete M. A. Austin A. (2020). Insight Crime: Balance de homicidios en las capitales de América Latina en 2019. Available online at: https://es.insightcrime.org/noticias/analisis/balance-homicid%20ios-capitales-2019/ (accessed October, 2022).

[B41] Ogilvie J. M. Stewart A. L. Chan R. C. Shum D. H. (2011). Neuropsychological measures of executive function and antisocial behavior: a meta-analysis. Criminology 49, 1063–1107. 10.1111/j.1745-9125.2011.00252.x

[B42] Page M. J. McKenzie J. E. Bossuyt P. M. Boutron I. Hoffmann T. C. Mulrow C. D. . (2021). The PRISMA 2020 statement: an updated guideline for reporting systematic reviews. J. Clin. Epidemiol. 134, 178–189. 10.1016/j.jclinepi.2021.03.001 33789819

[B43] Perestelo-Pérez L. (2013). Standards on how to develop and report systematic reviews in psychology and health. Int. J. Clin. Health Psychol. 13, 49–57. 10.1016/S1697-2600(13)70007-3

[B44] Pulido A. Ballén M. Quiroga L. A. (2017). Funciones ejecutivas, rasgos de personalidad e impulsividad en condenados por acceso carnal violento. Diversitas Perspectivas en Psicología 13, 169–185. 10.15332/s1794-9998.2017.0002.03

[B45] Richter J. J. (2010). ¿Es la cárcel el castigo más acorde a nuestros tiempos? Derecho y Humanidades 1, 279–291. 10.5354/0719-2517.2010.16016

[B46] Rocha N. Fonseca D. Marques A. Rocha S. Hoaken P. (2014). Cognitive function is associated with prison behaviour among women in prison but not with subjective perception of adjustment to prison. Crim. Behav. Ment. Health. 25, 389–402. 10.1002/cbm.1937 25251039

[B47] Rodriguez M. Boyce P. Hodges J. (2017). A neuropsychological study of older adult first-time sex offenders. Neurocase 23, 154–161. 10.1080/13554794.2017.1334802 28613138

[B48] Rodriguez M. Ellis A. (2018). The neuropsychological function of older first-time child exploitation material offenders: a pilot study. Int. J. Offend. Therapy Compar. Criminol. 62, 2357–2373. 10.1177/0306624X17703406 28397568

[B49] Romero-Martínez Á. Santirso F. Lila M. Comes-Fayos J. Moya-Albiol L. (2022). Cognitive flexibility and reaction time improvements after cognitive training designed for men perpetrators of intimate partner violence: results of a pilot randomized controlled trial. J. Fam. Viol. 37, 461–473. 10.1007/s10896-021-00304-2 34376906 PMC8339689

[B50] Santos-Barbosa M. F. Coelho-Monteiro L. M. (2008). Recurrent criminal behavior and executive dysfunction. Spanish J. Psychol. 11, 259–265. 10.1017/S1138741600004297 18630666

[B51] Schiffer B. Vonlaufen C. (2011). Executive dysfunctions in pedophilic and nonpedophilic child molesters. J. Sex. Med. 8, 1975–1984. 10.1111/j.1743-6109.2010.02140.x 21210954

[B52] Sedgwick O. Young S. Baumeister D. Greer B. Das M. Kumari V. (2017). Neuropsychology and emotion processing in violent individuals with antisocial personality disorder or schizophrenia: the same or different? A systematic review and meta-analysis. Aust. N. Zeal. J. Psych. 51, 1178–1197. 10.1177/0004867417731525 28992741

[B53] Seruca T. Silva C. F. (2015). Recidivist criminal behaviour and executive functions: a comparative study. J. Foren. Psych. Psychol. 26, 699–717. 10.1080/14789949.2015.1054856

[B54] Seruca T. Silva C. F. (2016). Executive functioning in criminal behavior: differentiating between types of crime and exploring the relation between shifting, inhibition, and anger. Int. J. Foren. Mental Health 15, 235–246. 10.1080/14999013.2016.1158755

[B55] Slotboom J. Hoppenbrouwers S. S. Bouman Y. Int Hout W. Sergiou C. van der Stigchel S. . (2017). Visual attention in violent offenders: susceptibility to distraction. Psych. Res. 251, 281–286. 10.1016/j.psychres.2017.02.031 28222312

[B56] Spenser A. Bull R. Betts L. Winder B. (2019). Executive functioning as a predictive measure of offending behaviour. J. Crim. Psychol. 9, 10–22. 10.1108/JCP-07-2018-0032

[B57] Tirapu-Ustárroz J. Cordero-Andrés P. Luna-Lario P. Hernáez-Goñi P. (2017). Propuesta de un modelo de funciones ejecutivas basado en análisis factoriales. Rev. Neurol. 64, 75–84. 10.33588/rn.6402.2016227 28075001

[B58] Vadini F. Calella G. Pieri A. Ricci E. Fulcheri M. Verrocchio M. C. . (2018). Neurocognitive impairment and suicide risk among prison inmates. J. Affect. Disord. 225, 273–277. 10.1016/j.jad.2017.08.030 28841492

[B59] Valizadeh S. Makvandi B. Bakhtiarpour S. Hafezi F. (2020). The Effectiveness of “Acceptance and Commitment Therapy”(ACT) on resilience and cognitive flexibility in prisoners. J. Health Promot. Manag. 9, 78–89.

[B60] Vilà-Balló A. Hdez-Lafuente P. Rostan C. Cunillera T. Rodriguez-Fornells A. (2014). Neurophysiological correlates of error monitoring and inhibitory processing in juvenile violent offenders. Biol. Psychol. 102, 141–152. 10.1016/j.biopsycho.2014.07.021 25108171

[B61] Viswanathan M. Berkman N. D. Dryden D. M. Hartling L. (2013). Assessing Risk of Bias and Confounding in Observational Studies of Interventions or Exposures: Further Development of the RTI Item Bank. Rockville: Agency for Healthcare Research and Quality. 24006553

[B62] Wallinius M. Nordholmd J. Wagnström F. Billstedt E. (2019). Cognitive functioning and aggressive antisocial behaviors in young violent offenders. Psych. Res. 272, 572–580. 10.1016/j.psychres.2018.12.140 30616126

[B63] World Prison Brief. (2020). World Prison Brief Data. Available online at: https://www.prisonstudies.org/world-prison-brief-data (accessed October, 2022).

[B64] Zou Z. Meng H. Ma Z. Deng W. Du L. Wang H. . (2013). Executive functioning deficits and childhood trauma in juvenile violent offenders in China. Psych. Res. 207, 218–224. 10.1016/j.psychres.2012.09.013 23036491

