# Supplementary material for: Alterations in executive functions in inmates convicted for violent behavior: a systematic review
Source: Front Psychol. 2023 Aug 16;14:1066474. doi: 10.3389/fpsyg.2023.1066474 (PMC10468974; doi:10.3389/fpsyg.2023.1066474)
Supplement: Supplementary file 1 [file Table_1.DOCX]

**Supplementary File 1**

**Database search strategy**

**1. PubMed**

("Prisoner"[TiAb] OR “criminal”[TiAb] OR “offender”[TiAb] OR “incarcerated”[TiAb]) AND ("Executive function"[TiAb] OR "executive dysfunction"[TiAb] OR “neurocognitive”[TiAb])

**2. Scopus**

TITLE-ABS-KEY ( "prisoner" OR "criminal" OR "offender" OR "incarcerated" ) AND TITLE-ABS-KEY ( "Executive function" OR "executive dysfunction" ) AND ( LIMIT-TO ( PUBSTAGE , "final" ) ) AND ( LIMIT-TO ( DOCTYPE , "ar" ) ) AND ( LIMIT-TO ( SUBJAREA , "PSYC" ) OR LIMIT-TO ( SUBJAREA , "NEUR" ) OR LIMIT-TO ( SUBJAREA , "SOCI" ) ) AND ( LIMIT-TO OR ( PUBYEAR , 2023 ) OR ( LIMIT-TO OR ( PUBYEAR , 2022 ) OR LIMIT-TO ( PUBYEAR , 2021 ) OR LIMIT-TO ( PUBYEAR , 2020 ) OR LIMIT-TO ( PUBYEAR , 2019 ) OR LIMIT-TO ( PUBYEAR , 2018 ) OR LIMIT-TO ( PUBYEAR , 2017 ) OR LIMIT-TO ( PUBYEAR , 2016 ) OR LIMIT-TO ( PUBYEAR , 2015 ) OR LIMIT-TO ( PUBYEAR , 2014 ) OR LIMIT-TO ( PUBYEAR , 2013 ) OR LIMIT-TO ( PUBYEAR , 2012 ) OR LIMIT-TO ( PUBYEAR , 2011 ) OR LIMIT-TO ( PUBYEAR , 2010 ) OR LIMIT-TO ( PUBYEAR , 2009 ) OR LIMIT-TO ( PUBYEAR , 2008 ) OR LIMIT-TO ( PUBYEAR , 2007 ) ) AND ( LIMIT-TO ( LANGUAGE , "English" ) OR LIMIT-TO ( LANGUAGE , "Spanish" ) )

**3. Web of Science**

(ALL=("prisoner" OR "criminal" OR "offender" OR "incarcerated") AND ALL=("Executive function" OR "executive dysfunction")) AND DOCUMENT TYPES: (Article) Refine by: PUBLICATION YEAR: (2023 OR 2022 OR 2021 OR 2020 OR 2019 OR 2018 OR 2017 OR 2016 OR 2015 OR 2014 OR 2013 OR 2012 OR 2011 OR 2010 OR 2009 OR 2008 OR 2007 ) AND DOCUMENT TYPES: ( ARTICLE ) AND LANGUAGES: ( ENGLISH ) AND RESEARCH AREAS: ( PSYCHOLOGY OR PSYCHIATRY OR NEUROSCIENCES NEUROLOGY OR BEHAVIORAL SCIENCES OR HEALTH CARE SCIENCES SERVICES OR SOCIAL SCIENCES OTHER TOPICS) Web of Science index=SCI-EXPANDED, SSCI, A&HCI, ESCI Timespan=2007-2023

**4. EBSCO**

("prisoner" OR "criminal" OR "offender" OR "incarcerated") AND ("Executive function" OR "executive dysfunction" OR "neuropsychological")

**5. EMBASE**

('prisoner'/exp OR 'prisoner' OR 'criminal'/exp OR 'criminal' OR 'offender'/exp OR 'offender' OR 'incarcerated') AND ('executive function'/exp OR 'executive function' OR 'executive dysfunction'/exp OR 'executive dysfunction') AND [article]/lim AND ([english]/lim OR [spanish]/lim) AND ([adult]/lim OR [young adult]/lim OR [middle aged]/lim OR [aged]/lim OR [very elderly]/lim) AND [humans]/lim AND [2007-2022]/py AND 'Article'/it

**Supplementary File 2**

**Adaptation of the RoB tool**

The procedure followed for the adaptation of this tool which included the seven questions of the original document:

1. Was the research question or objective in this paper clearly stated?

2. Was the study population clearly specified and defined?

4. Were all the subjects selected or recruited from the same or similar populations (including the same period)? Were inclusion and exclusion criteria for being in the study prespecified and applied uniformly to all participants?

5. Was a sample size justification, power description, or variance and effect estimates provided?

8. For exposures that can vary in amount or level, did the study examine different levels of the exposure as related to the outcome (e.g., categories of exposure, or exposure measured as continuous variable)?

11. Were the outcome measures (dependent variables) clearly defined, valid, reliable, and implemented consistently across all study participants?

14. Were key potential confounding variables measured and adjusted statistically for their impact on the relationship between exposure(s) and outcome(s)?

The last original question added: 13. Was loss to follow-up after baseline 20% or less? was changed to: The proportion of participants with missing data in the variable is irrelevant (or is adequately justified to be irrelevant) or it is justified that statistical techniques to deal with missing data are appropriate (e.g., weighting adjustments or imputation methods). The following questions were not considered during the risk of bias evaluation of the articles:

3. Was the participation rate of eligible persons at least 50%?

6. For the analyses in this paper, were the exposure(s) of interest measured prior to the outcome(s) being measured?

7. Was the timeframe sufficient so that one could reasonably expect to see an association between exposure and outcome if it existed?

9. Were the exposure measures (independent variables) clearly defined, valid, reliable, and implemented consistently across all study participants?

10. Was the exposure(s) assessed more than once over time?

12. Were the outcome assessors blinded to the exposure status of participants?

These adjustments were made since the information requested in the eliminated questions was not presented in most of the articles.
